# Is the soil quality monitoring an effective tool in consumers’ protection of agricultural crops from cadmium soil contamination?—a case of the Silesia region (Poland)

**DOI:** 10.1007/s10661-017-6413-5

**Published:** 2017-12-16

**Authors:** Agata Piekut, Renata Baranowska, Ewa Marchwińska-Wyrwał, Małgorzata Ćwieląg-Drabek, Ilona Hajok, Grzegorz Dziubanek, Elżbieta Grochowska-Niedworok

**Affiliations:** 1 Department of Environmental Health, School of Public Health in Bytom, Medical University of Silesia in Katowice (Poland), ul. Piekarska 18, 41-902 Bytom, Poland; 2 Department of Environmental Health Risk Factors, School of Public Health in Bytom, Medical University of Silesia in Katowice (Poland), ul. Piekarska 18, 41-902 Bytom, Poland; 3 Department of Human Nutrition, School of Public Health in Bytom, Medical University of Silesia in Katowice (Poland), ul. Jordana 19, 41-808 Zabrze, Poland

**Keywords:** Soil contamination, Plant contamination, Cadmium, Monitoring, Consumer health risks

## Abstract

The monitoring of soil quality should be a control tool used to reduce the adverse health effects arising from exposure to toxic chemicals in soil through cultivated crop absorption. The aim of the study was to evaluate the effectiveness of the monitoring and control system of soil quality in Poland, in terms of consumer safety, for agricultural plants cultivated in areas with known serious cadmium contamination, such as Silesia Province. To achieve the objective, the contents of cadmium in soils and vegetables in the Silesia administrative area were examined. The obtained results were compared with the results of soil contamination from the quality monitoring of arable soil in Poland. The studies show a significant exceedance of the permissible values of cadmium in soil samples and the vegetables cultivated on that soil. The threat to consumer health is a valid concern, although this threat was not indicated by the results of the national monitoring of soil quality. The results indicated an unequal distribution of risk to consumers resulting from contaminated soil. Moreover, the monitoring systems should be designed at the local or regional scale to guarantee the safety of consumers of edible plants cultivated in the areas contaminated with cadmium.

## Introduction

Most heavy metals enter the human body via food or by the consumption of edibles grown on contaminated soil. This route is the main source of a population’s exposure to hazardous substances such as cadmium, lead, and zinc (Hartwig 2013; Needleman et al. 1990). Studies conducted in this area indicate that approximately 60–80% of heavy metals, such as cadmium, present in the body of people living in industrialized areas have a dietary origin and is not the result of air pollution exposure (Bellows 1999; Oliver 1997; WHO 1999). Cadmium is relatively easily retrieved from the soil solution and transported to the aerial parts of the plants. Soil contamination by cadmium is therefore a potential risk to the food chain. It is an element that constantly accumulates in animal and human organisms. For plants, cadmium rises with increasing soil acidification (Bellows 1999; Cesar et al. 2011; Dziubanek et al. 2012; WHO 2010; Zhang et al. 2012). Considering the level of consumer exposure to cadmium, particular attention should be paid to the contamination of vegetables, the source of the greatest accumulation of this metal, both through the root system from the contaminated soil and as a result of atmospheric fallout on above-ground parts of the plant (Ociepa-Kubicka and Ociepa 2012; Wang et al. 2010). Food safety is an important factor affecting consumer health, and thus, it is essential for public health (WHO 2010).

Soil quality monitoring should be a control measure used to reduce the adverse health effects arising from exposure to toxic chemicals present in the edible plants cultivated on contaminated soils. In the European Union, there is no coherent policy for agricultural soil protection or a database of soil quality (EEA 2011). Some Member States, including Poland, have regulations in this regard. In Poland, for example, systematic soil quality controls are carried out across the country by an institution appointed for this purpose.

The source of soil quality data in Poland is the State Environmental Monitoring (SEM). Coordinated by the Chief Inspectorate of Environmental Protection (CIEP), it is an institutionalized system of data management regarding the state of the environment that primarily enables environment quality control through the analysis of specific parameters for each given component of the environment, including soil, and a compliance assessment with the normative requirements (CIEP 2012; CIEP 2011).

The monitoring includes soil at 216 selected, permanent monitoring sites located on agricultural soil throughout the country. The aim of the SEM is to assess the pollution level and changes in the soil’s characteristics in temporal and spatial dimensions. Approximately 40 physicochemical parameters are determined in the samples collected at designated soil profile sites: grain size composition (8 fractions), percent of humus, percent of CaCO_3_, pH, hydrolytic acidity, exchangeable acidity, the contents of plant-assimilable forms of phosphorous (P_2_O_5_), potassium (K_2_O), magnesium (Mg), and sulfur (S-SO_4_). Included are the contents of total nitrogen, organic carbon, polycyclic aromatic hydrocarbons, pesticides including organochlorine pesticides, exchangeable calcium, potassium, magnesium and sodium, electric conductivity, and radioactivity. Also included are the contents of soluble forms of calcium, magnesium, potassium, sodium, aluminum, iron, phosphorous, manganese, cadmium, copper, chromium, nickel, lead, zinc, cobalt, vanadium, lithium, beryllium, boron, strontium, and lanthanum. The aim of the monitoring of the chemistry of Poland’s arable soil is to track changes in the various characteristics of the soil used for agricultural purposes, particularly their chemical properties occurring in specific periods under the influence of agricultural and non-agricultural human activity. The assessment of soil pollution with heavy metals, including cadmium, sulfur, polycyclic aromatic hydrocarbons, and pesticides, is carried out in accordance with the Regulation of the Minister of the Environment concerning Soil and Land Quality Standards (Journal of Laws of 2016, item 1395) (Regulation of the Minister of Environment 2016).

The purpose of soil quality monitoring is to track changes in the features and characteristics of agricultural soil, especially the chemical properties of soil occurring at specific time intervals under the influence of agricultural and non-agricultural human activity (anthropopressure) (IUNG 2012). Collected data should help in the selection of appropriate corrective actions, in case of harmful influence on human life or the environment. The data also permit corrective actions or the removal of the effects in existing conditions, as in the case of ecological hazards. Studies of soil quality in Poland have been conducted since 1995, in 5-year cycles, by the Institute of Soil Science and Plant Cultivation (IUNG), commissioned by the Environmental Protection Inspectorate. The Environmental Protection Law Act (2001) imposes an obligation to monitor the chemistry of cultivated soils throughout the country. The criteria for soil quality assessments are defined by the Regulation of the Minister of Environment dated 1 September 2016 on soil quality standards and ground quality standards (Journal of Laws 2016. item. 1395) (Regulation of the Minister of Environment 2016).

The aim of the study was to evaluate the effectiveness of the monitoring and control system of soil quality in Poland, in terms of consumer safety, for agricultural plants cultivated in areas considered the most contaminated by cadmium, such as Silesia Province.

## Material and methods

In the study, to effectively assess arable soil quality monitoring in Poland, concentrations of cadmium in soil and vegetable samples were examined in the most contaminated area, Silesia Province. Data collected during the monitoring of the chemistry of arable soils in Poland carried out by the CIEP were taken from their report for the years 2010–2012 (IUNG 2012). Our results, obtained in the study, were compared with the results from the arable soil quality monitoring in Poland.

### Study area and sampling sites

The long-term operation of industrial plants involved in the mining and processing of non-ferrous metal ores, located in the Silesia administrative area and in the Province of Lower Silesia, makes these areas vulnerable to significant soil contamination by heavy metals. Heavy metals are characterized by very long periods of decomposition in soil, reaching up to several hundred years. Exposure to cadmium is a major health risk factor.

Soil and vegetable sampling points were located in different parts of the Silesia administrative area. Samples of edible plants were collected from arable soils in 24 territorial units of the Silesia Province. The study material consisted of 494 soil samples from agricultural fields and 90 samples of edible plants, including 23 samples of carrots, 17 samples of cabbage, 22 samples of parsley root, 15 samples of celery, and 13 samples of potato. The samples came from arable fields located on the outskirts of seven cities and two counties, near fixed points included in the state monitoring network. Both sorts of samples, soil and edible plants, were collected using the random sampling method.

Soil samples were collected from the stands with the surface of 1 m^2^. Between 15 and 20 individual samples were collected from each stand. The samples were taken from holes with a depth of 20 cm by using Egner’s soil sampler and were then mixed to form an aggregate sample representative of the given research stand.

### Sample preparation and chemical analysis

The soil samples were dried with the WG-71 Chemland dryer (Poland) at a temperature of 105 °C. Dried soil was sifted through a sieve with a diameter of < 2 mm. Afterward, the excess, with a weight of approximately 0.5 g, was formulated using the AS 60/220/D/2 Radwag analytical balance (Poland).

The vegetable samples were prepared for extraction the same way they are prepared for consumption: some non-edible parts of the vegetables were removed, and then they were washed, peeled, and shredded. Then, the excess matter of 1 g was created from each vegetable.

Soil and vegetable samples were subjected to extraction with the nitric acid (Merck) in a Teflon vessel using the microwave extraction method in the microwave reactor, model Magnum II, Ertec (Poland), with pressure and temperature computer control. Excess samples of dried soil underwent the extraction process, during which time they were digested by 8 mL of nitric acid in 10 min, and the excess samples of fresh vegetables were digested by 6 mL of nitric acid within 7 min. The pressure range amounted to 42–45 bar.

The content of cadmium in soil samples and edible plants was determined by inductively coupled plasma optical emission spectrometry (ICP-OES) (Integra XL, GBC spectrometer, Australia), LOQ = 0.01, LOD = 0.05. To examine vegetable samples with low cadmium content, the atomic absorption spectrophotometer (AAS) was used (SavantAA Sigma, GF 3000, GBC graphite furnace, Australia), LOQ = 0.5, LOD = 0.25. The result was the mean value obtained from three replicates. Cadmium concentration within the soil samples was calculated by dry weight, and in the case of edible plants by fresh matter. The result was the mean value obtained from three replicates. Cadmium concentration within the soil samples was calculated in conversion on dry matter of the soil, and in the case of edible plants on fresh matter. To create the calibration curve, the Certificate of Reference Material 1000 mg l^−1^ Cadmium Matrix, 2% HNO_3_ SPEX CertiPrep, was used. To confirm the accuracy of the performed analytical measurements, the following certified reference material was used in the soil samples: Analytical Reference Material Soil S-1, prepared by the Department of Physics and Nuclear Technology at the Academy of Mining and Metallurgy in Cracow. In the case of edible plants, the certified reference material was used (Certificate of Certified Reference Materials NCS ZC73012 Cabbage) from the China National Analysis Center for Iron and Steel.

## Results and discussion

### Monitoring of the chemistry of arable soil in Poland

**Fig. 1:**
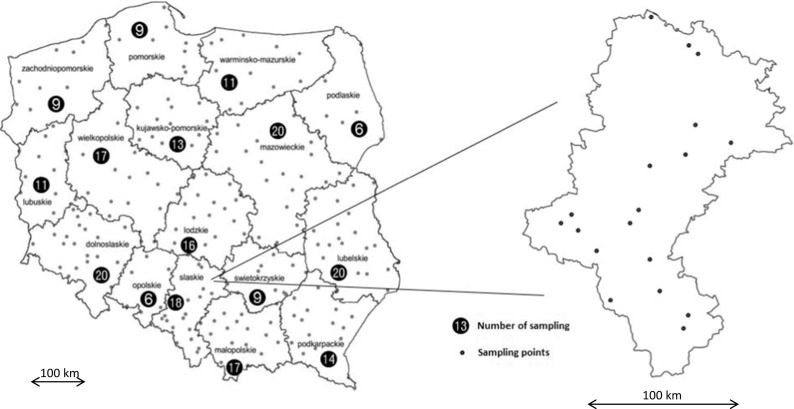
The location and number of IUNG measurement and control points in Polish provinces (IUNG 2012)

As a part of soil quality monitoring in Poland, soil samples retrieved from 216 fixed points of measurement and control, located across the country, were systematically examined (Fig. 1) (IUNG 2012). These points were located in agricultural and non-agricultural areas impacted by human activity. In the evaluation of CIEP, samples were selected in such a way that they are representative of agricultural lands with varying degrees of agricultural production intensification (Fig. 1) (IUNG 2012).

The Upper and the Lower Silesia regions are areas where intensive industrial activity was conducted in the past. The result was a significant environmental burden of many harmful contaminants, including cadmium.

The number of measurement and control points designated under the monitoring of the chemistry of arable soil in Poland, located in Silesia and Lower Silesia provinces, is comparable to the Lublin or Mazovia provinces, which are not as contaminated by heavy metals (20 points in the Lower Silesia Province, 18 in the Silesia Province, 20 in the Lublin Province, 20 in the Mazovia Province) (Fig. 1). Additionally, the distribution of measurement and control points, in the case of the Silesia Province, does not include areas most polluted by heavy metals. Most of these points are located on the outskirts of the province, treating marginally the most contaminated areas of the Upper Silesian Industrial Region. This is even more important, because in this area, despite the industry location, large quantities of edible plants, which are supplied to the local market, are cultivated (Fig. 1).

The monitoring of the chemistry of arable soils in Poland, conducted by CIEP in the years 2010–2012, revealed no significant exceedances of cadmium content in most investigated soil. The median of cadmium content in the year 2010 amounted to 0.17 mg/kg dry weight (d.w.) and was slightly lower than in previous research periods. Ninety percent of samples contained cadmium at a level lower than 0.56 mg/kg, with the maximum permissible content equal to 3 mg Cd/kg d.w., according to the Regulation of the Minister of Environment on soil quality standards and ground quality standards (IUNG 2012; Regulation of the Minister of Environment 2016).

At two monitoring points, as in previous periods of measurement, more than the maximum permissible concentration of cadmium, according to the Regulation of the Minister of Environment, was found. Both points were located in the Silesia Province. An extremely high concentration of cadmium (57.5 mg Cd/kg d.w.) was measured in a sample taken from the Piekary Slaskie, a location that has sustained long-term impacts of the zinc and lead steel industry (IUNG 2012). These excesses could be associated with the industry impact, as well as with the nature of the bedrock of the soil. Based on the statistical analysis of monitoring results, a concentration increase of any analyzed metal—in the 15 years of monitoring—was not observed. In the vast majority, concentrations of cadmium assume the natural levels for uncontaminated soil (IUNG 2012).

### Results of our studies

In the study, 494 samples of arable soil, collected from the Upper Silesia Region, were analyzed. The concentrations of cadmium were determined in the soil samples (Table 1). The Regulation of the Minister of Environment dated 1 September 2016 on soil quality standards and ground quality standards in Poland (Journal of Laws 2016. item. 1395) (Regulation of the Minister of Environment 2016) includes the maximum normative values for heavy metals in soil collected from the external layer (depth to 0.25 m). The maximum value for cadmium is 3 mg/kg d.w.

**Table 1:** The concentration of cadmium in soils cultivated in the territorial units of the Silesia Province

City/county	*N*	Concentration of Cd [mg kg^−1^ of dry weight]				
		Min	Max	Mean value	Median	SD
Bielski County	25	0.50	2.52	0.63	0.50	0.47
Bytom	28	0.50	28.62	8.46	5.52	7.13
Chorzow	2	4.27	19.34	11.81	11.81	10.66
Czestochowa	43	0.50	4.92	1.06	0.50	1.20
Dabrowa Gornicza	12	0.50	5.74	3.20	3.84	1.91
Gliwice	2	0.50	0.50	0.50	0.50	0.00
Jastrzebie-Zdroj	8	0.50	0.50	0.50	0.50	0.00
Katowice	6	15.28	48.79	28.60	25.99	11.46
Mikolow County	56	0.50	3.11	0.98	0.98	0.50
Myslowice	61	0.50	6.73	3.00	2.88	1.49
Myszkow County	43	0.50	3.42	0.99	0.50	0.79
Piekary Slaskie	52	3.52	68.47	18.95	14.71	12.72
Ruda Slaska	12	4.10	9.12	5.21	4.76	1.43
Rybnik	1			0.50		
Siemianowice Slaskie	22	0.50	10.70	7.07	7.68	3.00
Swietochlowice	1			7.28		
Tarnowskie Gory County	60	0.50	227.55	9.25	5.12	29.35
Tychy	1			0.50		
Wodzislaw County	45	0.50	0.50	0.50	0.50	0.00
Zabrze	13	0.50	3.37	1.63	2.09	1.15
Zory	1			0.50		

Source: results of research conducted in the Department of Environmental Health, School of Public Health in Bytom, Medical University of Silesia in Katowice

Among the 494 soil samples collected from the cultivated fields, as many as 198 (40%) were characterized by a higher cadmium content than the maximum permissible concentration. The highest value of cadmium was marked in the soil sample from cultivated fields from Tarnowskie Gory County (Miasteczko Slaskie). At 227 mg Cd/kg d.w., it exceeded the maximum allowable concentration by almost 76 times. In cultivated soil, derived from Chorzow, Katowice, Piekary Slaskie, and Ruda Slaska, all collected samples (100%) contained an over-normative cadmium concentration (Table 1).

Studies conducted by Baranowska (2016) also showed serious cadmium contamination of soil samples taken from home gardens and family allotment gardens (FAG) in the Silesia Province. In nearly 42% of the soil samples taken from the home gardens, cadmium concentrations were higher than the highest acceptable concentration established for cadmium; in fact, the highest reported concentration exceeded the normative value by more than 22 times. Among all soil samples taken from the allotments, only 67% did not exceed 3 mg Cd/kg d.w.

Because more than 60% of the vegetable demand of Silesia’s inhabitants is provided by the local produce, the cadmium content in the most frequently consumed vegetables grown in this region was also examined (Table 2) (AMA 2014). From cultivated fields, home gardens and allotment gardens, in the selected cities and counties, 90 samples of vegetables such as carrots, cabbage, parsley root, celery, and potatoes were collected. The Commission Regulation (EU), No 488/2014 of 12 May 2014 amending Regulation (EC) No1881/2006 (Commission Regulation 2014), established the maximum allowable levels for certain contaminants in foodstuffs, including vegetables:

- carrot, parsley root, peeled potato 0.1 mg/kg of fresh matter (f.m.);
- cabbage, celery 0.2 mg/kg f.m.

**Table 2:** The content of cadmium in vegetables from selected territorial units of the Silesia Province

City/county	Carrot		Cabbage		Parsley root		Celery		Potato	
	*N*	Cd [mg/kg f.m.]	*N*	Cd [mg/kg f.m.]	*N*	Cd [mg/kg f.m.]	*N*	Cd [mg/kg f.m.]	*N*	Cd [mg/kg f.m.]
Chorzow	3	0.53 2.00 4.83	–	–	3	0.22 0.25 0.36	4	0.31 1.11 2.82 4.82	–	–
Dabrowa Gornicza	1	0.21	1	0.43	–	–	1	0.12	–	–
Jastrzebie-Zdroj	2	0.11 0.03	–	–	–	–	–	–	–	–
Myszkowski County	6	0.16 0.22 0.41 0.23 0.21 0.27	5	< 0.06 < 0.06 0.55 0.06 < 0.06	6	0.10 0.14 0.28 0.61 0.13 0.24	6	0.28 0.09 0.19 0.10 0.15 0.23	6	< 0.06 < 0.06 0.16 0.38 < 0.06 < 0.06
Piekary Slaskie	3	0.66 0.23 0.79	11	0.58 0.37 0.17 0.33 0.34 0.21 0.20 1.15 0.36 < 0.06 0.14	3	0.40 1.30 1.23	–	–	3	0.28 < 0.06 0.32
Ruda Slaska	3	0.15 0.21 0.15	–	–	4	0.07 < 0.50 0.54 < 0.50	4	0.59 0.79 0.68 0.66	1	0.50
Siemianowice Slaskie	1	0.35	–	–	3	0.28 0.30 0.32	–	–	–	–
Sosnowiec	3	0.19 0.09 0.21	–	–	3	0.07 0.35 0.44	–	–	3	0.06 0.07 0.30
Tarnowskie Gory County	1	0.01	–	–	–	–	–	–	–	–

Source: results of research conducted in the Department of Environmental Health, School of Public Health in Bytom, Medical University of Silesia in Katowice

Studies have shown that in 87% of the analyzed samples of carrots, the maximum allowable cadmium concentration was exceeded, ranging from 10 to 4730%. In 53% of the cabbage samples, the determined cadmium concentration exceeded the maximum permissible value. The highest cadmium concentrations were observed in samples taken from Piekary Slaskie. In the cabbage cultivated in this city, a threefold excess of cadmium was reported. The cadmium content in parsley root in only 14% (22 samples) corresponded to the standards, and the indicated concentrations exceeded standards from 30 to 1200%.

The highest value of cadmium, determined in a sample from Piekary Slaskie, amounted to 1.3 mg/kg f.m. The analysis of cadmium content in celery samples showed that in 77% cadmium at concentrations exceeding the maximum permissible value was determined. The highest concentration of cadmium exceeded the limit value 24-fold (4.82 mg/kg f.m.). Cadmium concentration in potatoes was determined in the range of < 0.01 to 0.5 mg/kg f.m., which means that in 46% of samples, the maximum permissible concentration of this metal was exceeded (Table 2).

Considering the above results, which show significantly higher levels of cadmium than are permitted, both in soil samples and in vegetable samples, and given that the results of the national monitoring of soil quality do not reflect the scale of the problem, whether the present monitoring of agricultural soils is a guarantee of safe crops for consumers of agricultural products should be reconsidered. In case of children from the Upper Silesia, significant role in exposure to heavy metals also plays soil ingestion which results from hand-to-mouth activities, which was indicated in the studies conducted by Kicinska (2016a, b). High health risk through soil particle ingestion is typical for children from heavy metal-polluted areas (Li et al. 2017; Pelfrêne et al. 2013), such as Upper Silesia Industrial Region (Niec et al. 2013).

Data collected in the framework of soil quality monitoring should help guarantee the quality of soil and the safety of the food grown on them. Moreover, an objective of environmental monitoring, including the monitoring of soil quality, is to inform state administration authorities and society about the quality of tested environment components, the possible exceedance of parameters, and areas connected with those exceedances and their causes (CIEP 2012). Therefore, the proper implementation of these tasks should be based on high-quality data that pinpoint the potential sources of danger. Monitoring soil quality, beyond the implementation of tasks enabling effective environmental management, should also serve as an early warning system of any irregularities and potential public health hazards. For this purpose, it is necessary to obtain representative data that would help confirm the degree of soil contamination in the entire region and in the most problematic and densely populated areas. Without reliable data detailing the size and extent of environmental contamination (including soil contamination) and its potential hazardous effects, it is impossible to conduct any effective action to reduce health risks. According to the results, which noted an unequal distribution of risk to consumers resulting from contaminated soils, the monitoring systems should be designed at the regional or even local scale to better guarantee the safety of consumers of edible plants cultivated in the areas contaminated by cadmium.

## Conclusions

1. The study showed over normative concentrations of cadmium in samples of arable soil (over 40% of the samples) from the Silesia Province and in vegetables (carrot—up to 87% of the samples) cultivated on that soil, which could be a significant risk factor for consumers of food crops grown locally.
2. The monitoring of the chemistry of arable soil in Poland does not correspond to the role of a system allowing a proper assessment of the quality of this component of the environment, especially in problematic areas, such as the Silesia Province.
3. The data collected in the framework of the monitoring of the chemistry of arable soil in Poland and the present form of sourcing them do not guarantee the safety of consumers of edible plants cultivated in areas contaminated by cadmium, since in all samples of vegetables presented in this study cadmium was found in values exceeding the permissible limit.
4. The results showed that, in the case of agricultural soil, the monitoring systems should be designed at the regional or local scale to assess the local pollution risks to the population.
